# Changes in chemical components and antitumor activity during the heating process of Fructus Arctii

**DOI:** 10.1080/13880209.2019.1616778

**Published:** 2019-07-11

**Authors:** Jing Hu, Yun Shi, Bing Yang, Zibo Dong, Xinxin Si, Kunming Qin

**Affiliations:** a Department of Pharmacy, People's Hospital of Macheng City, Macheng, PR China;; b Zhejiang Chinese Medical University, Hangzhou, PR China;; c Nanjing King-Friend Biochemical Pharmaceutical Co., Ltd, Nanjing, PR China;; d Pharmacy School, Huaihai Institute of Technology, Lianyungang, PR China

**Keywords:** Traditional Chinese medicine, stir-heating, multi-component, compatibility

## Abstract

**Context:** The dried fruits of *Arctium lappa* L. have been used in two forms in traditional Chinese medicine; crude and stir-heating Fructus Arctii. However, its processed product possesses better activity.

**Objective:** In this study, the chemical constituents of both crude and processed Fructus Arctii and their antiproliferative activities were evaluated.

**Materials and methods:** The seven main active components in crude and various processed Fructus Arctii were quantitatively determined using high-performance liquid chromatography (HPLC). According to the actual amount in crude and five processed samples, seven single components were combined as multi-component combinations with six different proportions. The antiproliferative activities of these compatibility component groups were examined using the CCK-8 assay.

**Results:** During the heating process, the proportion of the seven main components changed dynamically. The contents of 3-caffeoylquinic acid (3-CQA), 3,5-dicaffeoylquinic acid (3,5-diCQA), and arctiin (ARC) declined, while the contents of 4-caffeoylquinic acid (4-CQA), 3,4-dicaffeoylquinic acid (3,4-diCQA), 4,5-dicaffeoylquinic acid (4,5-diCQA), and arctigenin (ARG) increased very significantly.

**Discussion and conclusions:** The results also indicated that seven components in the processed samples had higher cytotoxic profiles against HL-60 cells than those in the crude sample. Therefore, the heating process may enhance the antitumor activity of Fructus Arctii by changing the proportion of active components.

## Introduction

Traditional Chinese medicine (TCM) processing is a pharmaceutical technique that fulfills different requirements of therapy, dispensing, and making preparations (Zhao et al. 2010). Processing practices, which include stir-heating, charring, steaming, boiling, calcining, have been used to enhance the efficacy and/or reduce the toxicity of crude drugs (Cai 2009). The main mechanisms underlying processing are changes in the compositions of TCM drugs. Red ginseng is one of the representative examples of a processed herbal medicine. Heat processing can enhance the antitumor effects of ginseng by increasing the contents of ginsenosides Rg3, Rg5, and Rk1 (Wang et al. 2008; Choi et al. 2015; Park et al. 2016); it is generally thought that these changes in biological activity or alterations in components by processing can also occur in other TCM (Yoo et al. 2007).

Fructus Arctii, the dried fruit of *Arctium lappa* L., is commonly known as Niubangzi in China. It has widely been used to expel ‘wind-heat’, to relieve sore throat pain and swelling, and for detoxification (National Pharmacopoeia Committee 2015). Many kinds of components, such as lignans, phenols, fatty oils, and terpenoids, have been isolated from this plant (Qin et al. 2014). It is well known that seed TCM drugs should be stir-heated before clinical use (Hu et al. 2017a). In clinical application, the dried fruit of *A. lappa* is always stir-heated to lessen its ‘cold and smooth’ nature and to prevent it from impairing the spleen and stomach. In recent years, the heating process has been reported to affect the chemical components and efficacy of Fructus Arctii (Qin et al. 2015; Hu et al. 2017b).

**Figure 1.**
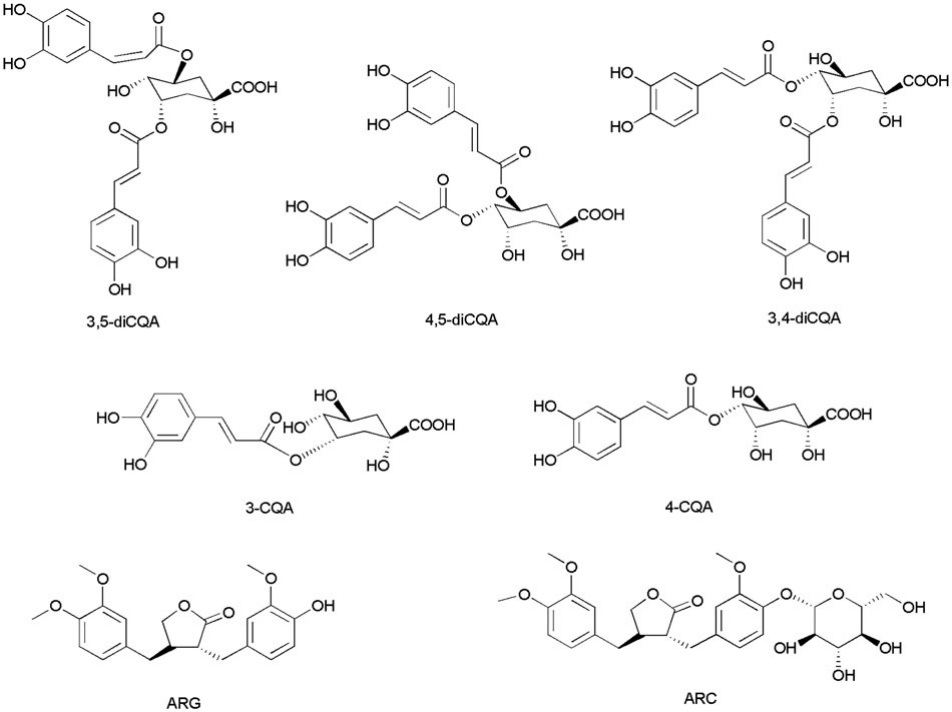
Chemical structures of seven main active components of Fructus Arctii.

We have previously identified six components, 3-CQA, 3, 5-diCQA, 3, 4-diCQA, 4, 5-diCQA, ARC, and ARG, as the main characteristic chemicals between crude and processed Fructus Arctii (Hu et al. 2017a, 2017b). Furthermore, another component was identified as 4-CQA by comparing the retention time with that of the corresponding standard substance, as well as the results of ultra-performance liquid chromatography coupled with quadrupole/time-of-flight mass spectrometry (UPLC-Q-TOF-MS/MS). 3-CQA, 4-CQA, 3, 4-diCQA, 4, 5-diCQA, and 3, 5-diCQA are isomers of caffeoylquinic acid derivatives, some of which have been reported to exhibit antioxidative activities and to induce granulocytic differentiation in HL-60 cells (Maruta et al. 1995; Mishima et al. 2005; Kim et al. 2011; Hwang et al. 2014). The structures of the seven components are shown in Figure 1. Lignans, such as ARC and ARG isolated from Fructus Arctii, show inhibitory activity and cytotoxicity against prostate cancer cell lines and HL-60 cells (Matsumoto et al. 2006; Ming et al. 2008). In addition, the ethanol extract of crude Fructus Arctii can inhibit the proliferation of colon cancer cells (HT-29), human rectal cancer cells (HRT-18), and human hepatoma cells (HepG2) (Zheng 2003). Moreover, *A. lappa* is one of the crucial herbs in herbal mixtures (e.g., ESSIAC^TM^ and Flor-Essence^TM^) that are sold as nutritional supplements and used to treat chronic conditions, especially cancer (Ferracane et al. 2010).

The chemical constituents of TCM drugs are the material bases for their effects, since stir-heating contributes to the chemical changes of Fructus Arctii. It is, thus, of great significance to investigate the relationship between the chemical constituents and antitumor effects of Fructus Arctii, aiming to clarify the mechanism of stir-heating and to guide rational processing in clinical practice. We, herein, postulated that different proportions of these seven components led to the changes of antitumor effect during the heating process. The contents of 3-CQA, 4-CQA, 3, 4-diCQA, 4, 5-diCQA, 3, 5-diCQA, ARC, and ARG during the processing of Fructus Arctii were determined by HPLC, then, the seven components were combined according to the contents in crude and various processed Fructus Arctii samples to evaluate the antitumor activity against HL-60 cells.

## Materials and methods

### Chemicals and reagents

DMSO was obtained from Sigma (St. Louis, MO, USA), penicillin/streptomycin solutions were purchased from Nanjing KeyGen Biotech Co., Ltd. (Nanjing, China), IMDM and FBS were acquired from GIBCO Life Technology (Grand Island, NY, USA), and cell counting kit-8 (CCK-8) was purchased from BIOSHARP (China). 3-CQA, 4-CQA, 3, 4-diCQA, 4, 5-diCQA and 3, 5-diCQA were obtained from Nanjing SenBeiJia Biotechnology Co., Ltd. (Nanjing, China) and ARC was obtained from Tianjin Chroma-standard Medical Science & Technology Development Co., Ltd. (Tianjin, China), while ARG was isolated and purified in our laboratory by column chromatographic method; its structure was characterized by NMR, UV, and mass spectroscopies, with the purity >98%. HPLC-grade methanol and acetonitrile were purchased from Tedia (Fairfield, USA), HPLC-grade formic acid was purchased from Nanjing Nanao Chemical Factory (Nanjing, China), and purified water was obtained from a Milli-Q water purification system (Millipore Corporation, Bedford, MA, USA). Other chemicals used were all of analytical grade.

### Preparation of standard substances

Stock solutions of the seven referenced components used for HPLC analysis were accurately weighed, dissolved in methanol in a volumetric flask, and diluted to appropriate concentration. A set of standard solutions with six different concentrations were prepared by further diluting the stock solution with methanol for the assessment of linearity.

Stock solutions for cell treatment were prepared in DMSO and stored at −20 °C. For all experiments, test compounds at final concentrations were freshly prepared by diluting the stock solution with phosphate-buffered saline.

### Preparation of crude and processed samples

Crude Fructus Arctii sample was collected from Nanjing Haichang Chinese Medicine Group Corporation (Nanjing, China) and authenticated by Professor Jianwei Chen (Nanjing University of Chinese Medicine, Nanjing, China); according to the Chinese Pharmacopeia 2015 edition, part one, the content of arctiin should not be lower than 5%. Processed samples were prepared in an oven by maintaining the oven temperature at 150 °C and heating for 3, 5, 10, 15, and 20 min. The samples with different processing degrees are the bases of the clinical application of TCM drugs, the processing experience of apothecary, and the research foundation of preliminary processing technology. The processed samples were later ground and treated in the same way as the crude samples. The extraction process was as follows: (i) the pulverized samples (100 g) were extracted with 70% ethanol under reflux (0.8 L, 1 h, three times); (ii) the extract solutions were filtered through a four-layer mesh, evaporated under a vacuum, and lyophilized.

### HPLC analyses

Analysis was conducted on a Shimadzu HPLC system that consisted of a quaternary pump, a DAD, and a column oven (Tokyo, Japan). Separation was performed on a YMC-Pack C18 column (250 mm × 4.6 mm, 5 µm) maintained at 35 °C. The mobile phase consisted of aqueous formic acid (0.01%, v/v) (A) and acetonitrile (B) using a linear gradient elution program as follows: 5–15% B at 0–20 min, 15–35% B at 20–75 min, and 35–50% B at 75–90 min. The flow rate was 1 mL/min, and the injection volume was 10 µL. Detection wavelength was set at 286 nm. The contents of the seven components in each sample were calculated using standard curves (Table 1). The moisture contents of crude and processed samples were determined according to guidelines provided by the Chinese Pharmacopeia 2015 edition. Results were obtained by deducting the moisture content and expressed as averages of duplicate analyses. The HPLC method was validated for linearity, precision, accuracy, stability, as well as recovery tests. All the validations were carried out under the regulation of the Chinese Pharmacopeia Commission (Part 4, appendix 9101), and the analysis was in accordance with the requirement.

**Table 1.** Linearities, correlation coefficients, LODs, and LOQs for determined components.

Component	Regression equation	*R* ^2^	Linear range (µg/mL)	LOD (µg/mL)	LOQ (µg/mL)
3-CQA	*y* = 18302*x* – 29479	0.9995	3.14-200.80	0.025	0.080
4-CQA	*y* = 18102*x* – 15313	1.0000	1.64-174.72	0.028	0.085
3,4-diCQA	*y* = 20297*x* – 33648	0.9999	1.75-122.00	0.079	0.243
3,5-diCQA	*y* = 21804*x* –79452	1.0000	10.26-513.00	0.085	0.298
4,5-diCQA	*y* = 23005*x* – 63821	0.9999	5.48-274.00	0.103	0.310
ARC	*y* = 3709.9*x* + 17034	0.9992	44.62-1428.00	0.500	1.510
ARG	*y* = 6984.4*x* – 881.05	0.9994	6.41-205.20	0.350	1.100

*y*: Peak area; *x*: Concentration (µg/mL).

### Antiproliferative effect on HL-60 cells

HL-60 cells were purchased from Nanjing KeyGen Biotech Co., Ltd. (Nanjing, China) and maintained in IMDM containing 20% FBS supplemented with 100 units/mL penicillin and 100 μg/mL streptomycin in a 37 °C incubator supplied with 5% CO_2_. Inhibition of cell proliferation was measured using the CCK-8 assay. Crude and five processed Fructus Arctii extracts were dissolved in DMSO. The content of each component in each group was the actual amount in crude sample or five processed samples. The seven components were eventually combined as compatibility component groups with six different proportions. Each component was accurately weighed according to the data in Table 2 and dissolved in 1 mL of DMSO. We determined the final concentration of each compatibility component group as 1 g/mL and diluted it to a specified concentration.

**Table 2:** Contents of seven components in Fructus Arctii during processing

	Content (mg/g)						
	3-CQA	4-CQA	3,4-diCQA	3,5-diCQA	4,5-diCQA	ARC	ARG
0 min	7.016	0.125	0.255	6.766	1.040	71.816	2.125
3 min	6.576	0.810	1.136	2.782	4.534	75.780	2.152
5 min	5.894	0.794	1.144	2.535	4.220	67.553	2.073
10 min	5.351	0.961	1.261	2.199	3.853	67.411	2.253
15 min	4.894	0.995	1.224	1.976	3.492	65.077	2.670
20 min	4.519	1.041	1.226	1.817	3.149	64.821	3.517

HL-60 cells were seeded in 96-well plates at a density of 1.0 × 10^5^/well with 10% FBS and cultured. After 24 h of culture, various concentrations of extracts/compatibility component groups were added to the wells and incubated for 72 h. Then 10 μL of CCK-8 was then added to each well and the mixture incubated at 37 °C for another 3 h. Measurements were carried out at 450 nm by a microplate reader (BioTek Instruments). All tests were performed in triplicate, independently.

Proliferation rate (%) = (sample OD − blank OD)/(control OD − blank OD) × 100%.

### Statistical analysis

Results were analyzed using the SPSS 23.0 software program and data are represented as mean ± SD. The significance of difference was determined using one-way ANOVA and the post hoc Dunnett’s test. Differences were considered to be statistically significant when values of *P* < 0.05.

## Results

### Contents of seven components during heating

**Figure 2.**
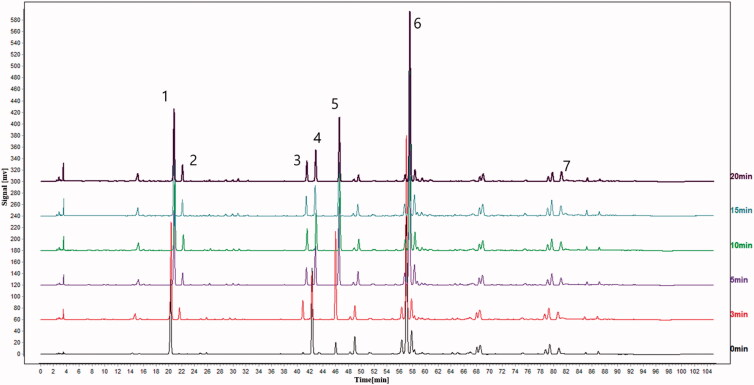
The HPLC chromatograms of crude Fructus Arctii and the different processed samples.

**Figure 3.**
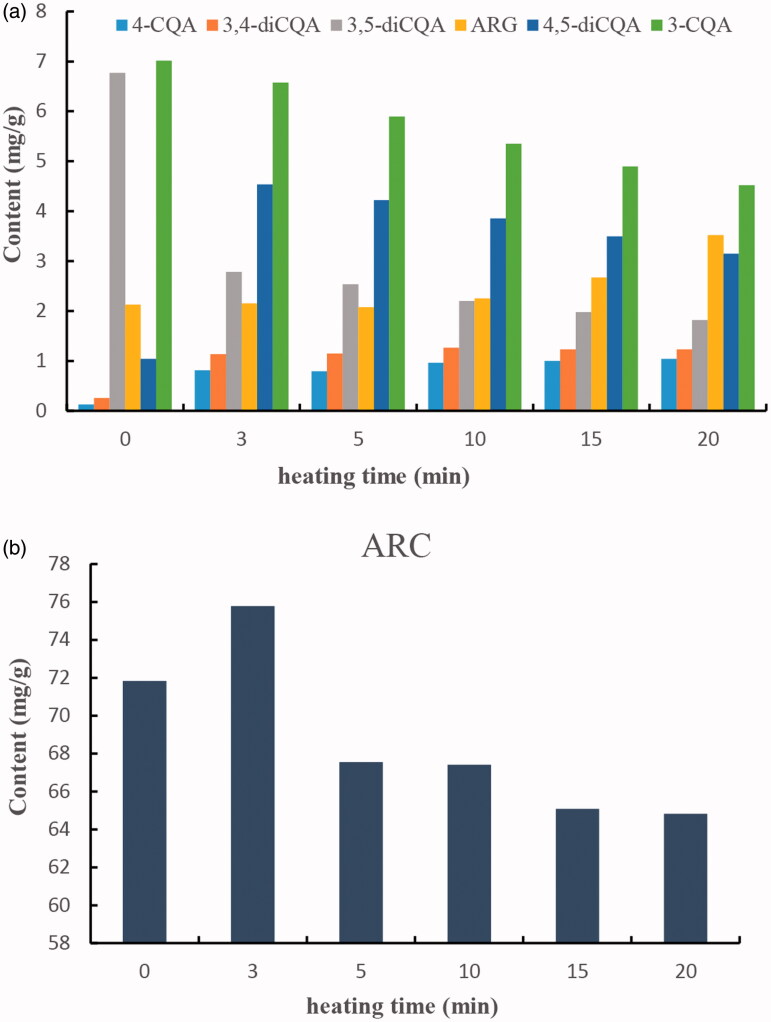
Contents of seven components in Fructus Arctii during processing. (A) 3-CQA, 4-CQA, 3,4-diCQA, 3,5-diCQA, 4,5-diCQA, ARG; (B) ARC.

An HPLC method was established to characterize and determine the main components in Fructus Arctii. The 7 main components were found and identified by comparing the retention time with corresponding standard substances. Under the HPLC conditions described above, the retention times of 3-CQA, 4-CQA, 3, 4-diCQA, 3, 5-diCQA, 4, 5-diCQA, ARC, and ARG were 20.22, 21.54, 40.81, 42.23, 45.93, 56.94, and 80.62 min, respectively. The HPLC chromatograms of crude and five processed samples are shown in Figure 2. During the heating process, the contents of the seven components changed significantly. The contents of ARC, 3-CQA, and 3, 5-diCQA reduced, whereas those of 4-CQA, 3, 4-diCQA, 4, 5-diCQA, and ARG increased. The content changes of these constituents are shown in Figure 3. ARC was the major component in both crude and processed samples, with its content change ranging from 75.780 mg/g to 64.821 mg/g. At the beginning of the heating process, the content of ARC rose from 71.816 mg/g to 75.780 mg/g, but then plummeted to 64.821 mg/g with prolonged heating time. During the heating process, the seed coat of Fructus Arctii splits open, which can promote the dissolution of ARC. However, the glycosidic linkage of ARC breaks down and transforms into ARG for high temperature. Collectively, the contents and proportions of the seven components in Fructus Arctii varied significantly during the heating process, which induced different antiproliferative effects. Further studies on antitumor activity were, therefore, carried out with HL-60 cells.

### Antiproliferative effects of compatibility component groups on HL-60 cells

**Figure 4.**
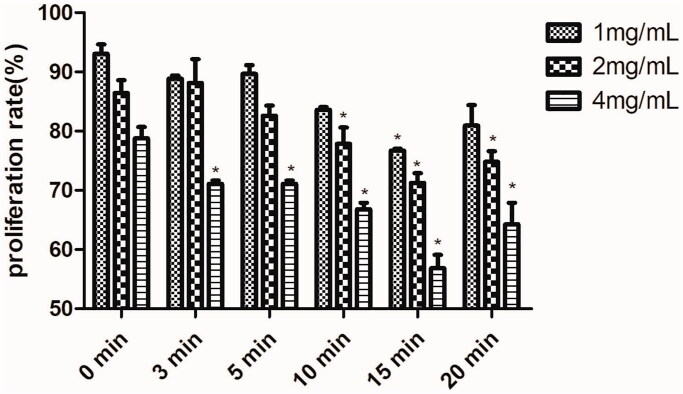
Effects of six compatibility component groups on proliferation of HL-60 cells after 72 h of treatment. Data are represented as means ± SD in three independent experiments. Significance: *p < 0.05 vs. compatibility component group at 0 min.

The effects of Fructus Arctii extracts/six compatibility component groups on the viability of HL-60 cells were examined using the CCK-8 assay and were recorded as proliferation rates. Antiproliferation results for HL-60 cells showed that crude and five processed Fructus Arctii samples had IC_50_ values of 318, 147, 144, 126, 114, and 93 μg/mL, respectively. Compared to the crude Fructus Arctii extract, the processed extracts significantly increased the antiproliferative activity on HL-60 cells. At 250 μg/mL, the crude extract inhibited HL-60 cell growth by 23.72%, whereas after stir-heating, the inhibition rates of the processed samples were 69.57 − 85.63%. Consequently, changes in antiproliferative effects of Fructus Arctii extracts may be related to the chemical constituents. As shown in Figure 4, different groups exert various inhibitory effects. Compared to the 0 min compatibility component group, 3 min, 5 min, 10 min, 15 min, and 20 min groups showed significant inhibitory effects. Moreover, the seven components in the processed samples had higher cytotoxic profiles against HL-60 cells than those in the crude sample, with the 15 min compatibility component group functioning most significantly. In the 15 min processing group, there are 3 subgroups, and in the 4 mg/mL subgroup, the concentration of each component was 19.57 μg/mL for 3-CQA, 3.98 μg/mL for 4-CQA, 4.90 μg/mL for 3, 4-diCQA, 7.90 μg/mL for 3, 5-diCQA, 13.97 μg/mL for 4, 5-diCQA, 260.31 μg/mL for ARC, and 10.68 μg/mL for ARG, and in the 2 mg/mL subgroup, the concentration of each component was halved, the cell viability decreased significantly (*p* < 0.001). Taken together, the proportions of 3-CQA, 4-CQA, 3, 4-diCQA, 3, 5-diCQA, 4, 5-diCQA, ARG, and ARC in the 15 min group were optimum. The antiproliferative effects were attenuated when the heating time exceeded 15 min.

## Discussion

To the best of our knowledge, the compatibility component group of Fructus Arctii is studied for the first time to compare the antitumor activities. The results indicated that constituent profile and the proportion of seven main components in Fructus Arctii changed significantly during heating process. The proportion of active components played an important role in the pharmacological effect of Fructus Arctii.

## Conclusions

In summary, heating process led to variation in the content and proportion of the components in Fructus Arctii, which might enhance the antitumor activity. The changes of pharmacological activity motivate us to further explore the relationship between the best compatibility proportion and the processing technology of Fructus Arctii.
